# Hesperidin Loaded on Gold Nanoparticles as a Drug Delivery System for a Successful Biocompatible, Anti-Cancer, Anti-Inflammatory and Phagocytosis Inducer Model

**DOI:** 10.1038/s41598-020-66419-6

**Published:** 2020-06-09

**Authors:** Ghassan M. Sulaiman, Hanaa M. Waheeb, Majid S. Jabir, Shaymaa H. Khazaal, Yaser Hassan Dewir, Yougasphree Naidoo

**Affiliations:** 1 Biotechnology Division, Applied Science Department, University of Technology, Baghdad, Iraq; 2 Applied Chemistry Division, Applied Science Department, University of Technology, Baghdad, Iraq; 3 King Saud University, College of Food and Agriculture Sciences, P.O. Box 2460, Riyadh, 11451 Saudi Arabia; 4 Kafrelsheikh University, Faculty of Agriculture, Kafr El-Sheikh, 33516 Egypt; 5 University of KwaZulu-Natal, School of Life Sciences, Westville Campus, Private Bag X54001, Durban, 4000 South Africa

**Keywords:** Biological techniques, Biotechnology, Cancer, Drug discovery

## Abstract

Hesperidin is a flavonoid glycoside with proven therapeutic activities for various diseases, including cancer. However, its poor solubility and bioavailability render it only slightly absorbed, requiring a delivery system to reach its therapeutic target. Hesperidin loaded on gold nanoparticles (Hsp-AuNPs) was prepared by a chemical synthesis method. Various characterization techniques such as UV-VIS spectroscopy, FTIR, XRD, FESEM, TEM and EDX, Zeta potential analysis, particle size analysis, were used to confirm the synthesis of Hsp-AuNPs. The cytotoxic effect of Hsp-AuNPs on human breast cancer cell line (MDA-MB-231) was assessed using MTT and crystal violet assays. The results revealed significant decrease in proliferation and inhibition of growth of the treated cells when compared with human normal breast epithelial cell line (HBL-100). Determination of apoptosis by fluorescence microscope was also performed using acridine orange-propidium iodide dual staining assay. The *in vivo* study was designed to evaluate the toxicity of Hsp-AuNPs in mice. The levels of hepatic and kidney functionality markers were assessed. No significant statistical differences were found for the tested indicators. Histological images of liver, spleen, lung and kidney showed no apparent damages and histopathological abnormalities after treatment with Hsp-AuNPs. Hsp-AuNPs ameliorated the functional activity of macrophages against Ehrlich ascites tumor cells-bearing mice. The production of the pro-inflammatory cytokines was also assessed in bone marrow–derived macrophage cells treated with Hsp-AuNPs. The results obviously demonstrated that Hsp-AuNPs treatment significantly inhibited the secretion of IL-1β, IL-6 and TNF.

## Introduction

The burden caused by cancer to the global public health has been markedly increasing, as evidenced by an estimate of 9.6 million mortalities in 2018 (around one sixth of the overall worldwide mortalities), around 70% of which being recorded in nations with low to middle income^1,2^. The most frequently diagnosed type of cancer in females is breast cancer, being the reason for more than one tenth of annual newly diagnosed cases and the second cause of mortality. In Iraq, breast cancer occupies the second rank, following cardiovascular diseases, as a major cause of mortality (23% of cancer-related deaths in women)^3^. Direct administration of the available chemical drugs is generally problematic, while these chemicals often exert toxicity and unwanted side effects to the patient’s tissues^4^. Other common modes of treatment such as surgical intervention and radiotherapy exert limited sufficiency, making it urgent to develop novel approaches to treat cancer^5^.

In order to selectively deliver drugs to the cancer lesion, several strategies have been developed. Most of these rely on unique biological and physical features of the tumor microenvironment such as leaky vasculature, compromised lymphatic drainage, angiogenesis, acidic pH, overexpression of cell membrane antigens^6^, which are exploited to gain access to the cancer cells. Recently, particular focus has been paid to the causal relationship between inflammation and cancer development^7^. The local immune response and systemic inflammation have important roles in tumor progression, as well as patient survival in established cancers^8^. The study of inflammatory pathways activated during cancer progression has paved the way for novel therapeutic strategies based on the use of nanoparticles. Nanomedicine has emerged as an appealing diagnostic tool and a novel therapeutic strategy for cancer treatment. Current developments involving the use of NPs hold promising potentials to reach highly efficient and safer approaches to deliver anticancer drugs to their target tissues^9^.

The unique characteristics of gold nanoparticle (AuNPs), along with their multiple surface functionalities, enhanced their broad applications in the field of bionanotechnology, particularly as drug-delivery systems since NPs are biocompatible and possess a surface are that is sufficiently high to load more elevated concentration of the delivered drug or even several drugs on one particle^10^. Through their role as drug carriers, gold NPs reportedly achieved superior results over the traditional cancer treatment, with continuously growing research on the best methods for their development and applications^11^. AuNPs is the most extensively investigated and promising metal NP known to deliver paclitaxel, a widely known anti-cancer drug. Au NPs designed and synthesized in different shapes and configurations as Au-nanoshells, Au-nanorods and Au-nanocages are now emerging as a versatile nanovehicle for cancer therapy^12^.

Long-term epidemiological research found that people who consume flavonoid-containing diets in a regular basis have lower risk of cancer and other chronic diseases^13^, leading to an enhanced interest in the use of these compounds as food supplements when managing cancer cases^14^. Hesperidin (3, 5, 7-trihydroxy-4–methoxy-flavanone-7-rhamnoglucoside) is a flavanone glycoside that is a member of the flavonoid family that demonstrated effective antioxidant and anticancer roles^15^. Hesperidin extracted in high amounts from citrus fruits was shown to possess roles against oxidative stress, inflammation, and hepyerglycemia^16^. Recent investigations also showed effective anti-oxidative, anti-obesity and anticancer function of hesperidin^17,18^. However, this compound has not been widely used clinically due to its decreased solubility in water, leading to turn the focus of research on overcoming this issue via different methods including the invention of sufficient delivery systems for hesperidin-containing drugs^19^. In our previous study we introduced a new method to enhance the drug’s efficiency, solubility, biocompatibility and cytotoxic effects on human breast MCF-7 cancer cells by synthesizing hesperidin encapsulated PLGA nanoparticles. Such models need to deliver the drug to the targeted tissue using a highly active system, which can be based on a variety of biomaterials including the biodegradable nanoparticles. To enhance the efficiency of the cancer therapeutic agent and its biocompatibility, combination therapy may be more effective than single agents. Thus, the loading of hesperidin on gold nanoparticles (Hesp-AuNPs) was performed in the present study, where their activity against oxidative stress along with their haemolytic effect to human red blood cells were investigated. In addition, an animal model bearing Ehrlich ascites tumor cells was employed to clarify the toxicological mechanisms of action, the immunomodulatory responses, and phagocytic activities. The cytotoxic potentials against human triple –negative breast cancer cell line MDA-MB-231 were also determined.

## Materials and Methods

### Materials and reagents

HAuCl_4_·3H2O was purchased from Strem Chemicals, Inc., (Newburyport, MA, USA). Reduced L-glutathione (GSH) was from Sigma-Aldrich Chemical Co., (St. Louis, MO, USA), while, sodium hydroxide (NaOH) was purchased from Merck (Germany). Acridine orange, ascorbic acid, ethidium bromide, fetal bovine serum, trypsin-EDTA, dimethyl sulfoxide (DMSO), 3-(4,5-dimethylthiazal-z-yl)-2,5-diphenylterazolium (MTT) and crystal violate were provided by Sigma Chemical Co. (St. Louis, MO, USA); Roswell Park Memorial Institute 1640 medium (RPMI) medium, fetal bovine serum, and lipopolysaccharide (LPS) were provided by Sigma -Aldrich (Germany). Contamination with microbes was prevented by penicillin and streptomycin (Biosource International, Nivelles, Belgium). All other chemicals and reagents were of analytical grade level.

### Cell lines

The human triple–negative breast cancer cell line MDA-MB-231 was provided by the Biotechnology Research Center, University of AL- Nahrain, Baghdad, Iraq. Ehrlich ascites tumor cells -bearing mice and in human normal breast epithelial cell line (HBL-100) were provided by Iraqi Center for Cancer and Medical Genetic Research, AL-Mustansiriyah University, Baghdad, Iraq. For *in vitro* cytotoxicity assay, cultures of MDA-MB-231 or HB-100 cells were made in tissue culture flasks (T 25 cm^2^; Falcon, USA) containing RPMI-1640 medium supplemented with 10% FBS, 2 mM L-glutamine, and 20 mM (5% CO_2_, 37 °C). To examine the activity of macrophages against tumor cells, Ehrlich ascites tumor cells were maintained under similar conditions of normal or breast cancer cells.

### Preparation of gold nanoparticles

Gold nanoparticle was synthesized by the method of Wu and his co-worker^20^. Briefly, an aqueous solution of tetrachloroauric acid (0.025 M of HAuCl_4_.3H_2_O, 1 mL) was mixed with an aqueous solution of GSH (0.019 M, 8 mL) and stirred vigorously for 30 min. Then, NaOH (0.1 M) was used to bring the pH of the mixture to 8. Immediately, the yellow color disappeared and after 30 min a colorless solution appeared. A freshly prepared solution of NaBH_4_ (2 mg mL^−1^ in water) was added drop by drop under vigorous stirring until the solution changed to red color. After that, the solution was stirred and left overnight at room temperature for further reaction. The excess GSH was eliminated by ultracentrifugation using a Vivaspin 20 column (13,600 rpm for 30 min) and sodium phosphate buffer at pH 8.0 (10 mM, ~10 mL × 20) to remove salts and free GSH. After centrifugation, the supernatant was removed and the deposited GSH-AuNPs were dissolved in 2 mL of deionized distilled water.

### Conjugation of hesperidin with gold nanoparticle

For the preparation of a nano-conjugated system, hesperidin (C_28_H_34_O_15_, 500 μg mL^−1^) was mixed with the prepared gold nanoparticles (AuNPs) overnight with continuous stirring at room temperature. The resulting AuNP-conjugated hesperidin (Hsp-AuNPs) was purified using ultracentrifugation (13.000 rpm, 30 min., 15 °C) to eliminate excess hesperidin. AuNPs loaded with the drug made up a loose pellet with an intense red color and they were employed for additional tests^21^.

### Characterization of prepared nanoparticles

FTIR analysis was achieved using an FTIR spectrometer (8400 S, Shimadzu, Japan) with attenuated total reflection mode, spectral range of 4000–400 cm^−1^, and a resolution of 4 cm^−1^. Measurement of the crystalline status of the prepared samples was performed by an X-ray diffractometer machine (XRD-6000, Shimadzu, Japan). A Cu Kα incident beam (λ = 1.542 A°) at 2θ = 20°− 60° was employed to obtain the diffraction patterns. X ray tubes had a voltage of 45 kV and a current of 30 mA. Field Emission Scanning Electron Microscopic (FESEM) analysis was conducted using MIRA 3 TESCAN (Brno -Czech Republic) coupled to an EDAX Team (EDAX, Mahwah, NJ 07430, USA) to examine the shape and presence of the prepared nanoparticles. Transmission electron microscope (TEM; Tecnai G2 20 S-TWIN, China) was applied to examine the shapes of the prepared nanoparticles. Zeta potentials and particle size were measured by Brookhaven Zeta PALS instrument (Milton Keynes, UK) to characterize the nanoparticles.

### *In vitro* release with different pH values

The release of hesperidin from the gold nanoparticles, on which it was loaded, as well as from the free hesperidin was performed *in vitro* as previously reported^22^, with minor modification. Briefly, the NPS-drug mixture was subjected to centrifugation (13.000 rpm, 30 minutes, 15 °C), the loose red pellet was taken and divided into several aliquots (200 μL). And subjected to dilution with 800 μL of either physiological (pH 7.4) or acidic (pH 5.04). Incubation of the mixtures was performed at 37 °C for different periods of time (5, 15, 30, 60, 120, 180, 240, 1440, and 2880 min). After each period, measurement of the absorbance of the solution was achieved by a UV-VIS spectrophotometer at 286 nm. The concentrations of the released Hsp from each period were recognized by using the following formula:

$${\rm{Release}}\,( \% )=({\rm{W1}}/{\rm{Wt}})\times 100$$

where W1 is the releasing weight of Hsp, while Wt is the loading weight of Hsp.

### Blood sampling and preparation

Fresh samples of blood were taken from 10 healthy donors and distributed into heparin-coated tubes based on the method of National Institute of Health and Food and Drug Administration and as per the declaration and regulation of Helsinki of 1975 as a statement of ethical principles. Permission was obtained from the hospitals of the medical city in Baghdad, Iraq and approved by the institutional ethical committee of university of technology, Baghdad, Iraq (Ref. No. AS 13–7–01–2019). The study participants were informed about the value of the study before we are gaining to collect any data or samples. Informed consent and/or assent were obtained from the study participants.

### Hemolysis test

Hemolysis test was performed for the prepared Hsp, AuNPs and Hsp-AuNPs samples based on a previously described method^23^, with some modifications. Briefly, whole blood (100 µL) was mixed with PBS (700 µL) and 100 µL of Hsp, AuNPs or Hsp-AuNPs at five concentrations (20, 80, 160, 240 and 320 µg mL^−1^) were added; phosphate buffer saline served as a negative control (0% hemolysis), whereas deionized distilled water served as a positive control (100% hemolysis). Triplicate samples were employed, which were subjected to incubation (37 °C, 1 h). Eventually, centrifugation of all mixtures was achieved (700 rpm, 5 min) and absorbance was read (541 nm, UV–Vis spectrophotometer). Hemolysis value was extracted as follows:

$${\rm{Percentage}}\,{\rm{hemolysis}}={\rm{OD}}\,{\rm{sample}}-{\rm{OD}}\,(-{\rm{ve}})\,{\rm{control}}/{\rm{OD}}\,(\,+\,{\rm{ve}})\,{\rm{control}}-{\rm{OD}}\,(\,-\,{\rm{ve}})\,{\rm{control}}$$

Next, optical microscopy was employed to further examine the samples

### *In* vivo assays

#### Laboratory mice

Male BALB/c mice were obtained from the Iraqi Center for Cancer and Medical Genetic Researches, University of Al-Mustansiriyah, Baghdad, Iraq. The mice, with an age range of 5 to 6 weeks and weighing 25–30 g, were housed at a temperature of 24 ± 2 °C and a humidity of 55 ± 10% on a 12:12 hours light/dark cycle with full supply of water and food. All procedures were performed in accordance with the U.S. National Institutes of Health (NIH) Guide for the Care and Use of Laboratory Animals (NIH Publication No. 86–23, revised in 1996) and were approved by Animal Care and Ethics Committee at Biotechnology Division, Applied Sciences Department, University of Technology, Baghdad, Iraq.

### Toxicity assay

A random categorization of the 15 mice used in the present study was adopted; 2 test groups with various doses of Hsp-AuNPs as well as a control group. Mice were intraperitoneally injected with Hsp-AuNPs solution (about 100 µL; the volume was adjusted according to animal weight for each dose) at doses of 20 and 200 mg kg^−1^ for three times per week (every second day). The control group was injected with saline. Body weight and behavior of mice were accurately documented prior to the injections and throughout the experiment’s duration. Mice of each group were then sacrificed following the final treatment at day 7 and day 14 and several organs (liver, spleen, kidney and lungs) were immediately excised immediately. Blood was collected by cardiac puncture and serum was collected after (3000 rpm, 10 min). For histological examination, the selected organs were subjected to steps of washing (PBS), fixation (10% formalin) and embedding with paraffin following the paraffin dispensing module EG 1150 H (Leica, Germany). Samples were sectioned (microtome RM2255, Leica, Germany) and stained with hematoxylin and eosin.

### Cytotoxicity against cell lines

Seeding of MDA-MB-231 or HB-100 cells (200 μL, 1 × 10^5^ cells mL^−1^) was performed using 96-well flat-bottom culture plates (Falcon, USA). Following 48 h of exponential growing, the cells were incubated with Hsp, AuNPs and Hsp-AuNPs at concentrations of 0, 25, 50, 75, 100 and 125 (µg mL^−1^, 24 h). After that, a mixture of MTT stain and PBS (50 µL) was applied to the wells and the cells were incubated (10–15 min, 37 °C). After discarding the stain, a washing with tap water was performed and isopropanol (100 µL) was applied for dissolving the stain. The cells were then incubated (10 min) to eliminate the bubbles and the absorbance was read (492 nm; microplate reader, ELx 800, Bio-Tek Instruments Inc., USA). The equation below was applied to calculate the inhibition value:

$${\rm{Inhibition}}\,{\rm{rate}}\,( \% )=({\rm{A}}-{\rm{B}}/{\rm{C}})\times 100$$

where A is the optical density of the control while B is that for the sample.

For more confirmation that growth of MDA-MB-231 cells was inhibited through induction by Hsp, AuNPs and Hsp-AuNPs, *in vitro* experiments were performed. Briefly, seeding of the cells (200 μL, 1 × 10^6^ cells mL^−1^) was conducted on sterile coverslip within culture plates (24-well flat-bottom). Following 48 h of exponential growth, the cells were incubated (24 h) with the IC_50_ of the tested compounds. Thereafter, the cells were stained with a mixture of crystal violet and PBS (50 µL) and incubated (10–15 min, 37 °C). After discarding the stain and washing with tap water, the coverslips were dried and subjected to photography.

### Acridine orange and propidium iodide staining

The Hsp-AuNPs induced death of MDA-MB-231cells was tested by applying double staining with acridine orange and propidium iodide^24^. Briefly, the cells were placed in 96-well plates, followed by incubation with the IC_50_ concentration of Hsp, AuNPs and Hsp-AuNPs (16 h). After detachment and washing for two times with PBS, the cells were moved into a clear 96-well plate. Fluorescent staining with 10 µL of acridine orange (1 mg mL^−1^) and propidium iodide (1 mg mL^−1^) was then performed following by visualization with a fluorescence microscope.

### Macrophages activity to tumor cells

The macrophage function in the peritoneal cavity was determined according to method of Orsolic and Basic^25^. Briefly, the Hsp-AuNPs were given at a dose of 20 mg kg^−1^ three days a week and Ehrlich cancer cells were inoculated after 2 hours (2 × 10^6^ mouse ^−1^). The positive and negative control groups were also included. Mice were sacrificed on day seven the external abdominal region was disinfected, and inoculation with 3 ml of saline in the peritoneal membrane was conducted. After collecting the peritoneal cells-containing solution, fixation of the cells was performed using glutaraldehyde (2.5%), followed by staining with Giemsa stain (5%) and photographing (×400) using phase-contrast microscopy.

### Bone marrow-derived macrophages

Bone marrow-derived macrophages (BMDMs) were isolated from female BALB/c mice (aged 7–8 weeks) as described by Celada *et al*.^26^. Briefly, cells were cultured in RPMI 1640 supplemented with 10% heat inactivated fetal bovine serum, streptomycin 100 IU/ml, penicillin 100 IU/ml, 2 mM L-glutamine, and 25 mM HEPES (pH 7.3). Macrophages were selected by colony stimulating factor 1 (macrophage) (PeproTech, 315–02) 10 ng/ml. Cells were allowed to grow for between 6 to 9 days before use.

### IL-6, IL-1 β and TNF-α

Brown marrow derivative macrophage cells were treated with 50 or 250 µg mL^−1^ of Hsp-AuNPs. As a positive control, 1 µg mL^−1^ lipopolysaccharide (LPS) and Adenosine triphosphate (ATP) was used to activate control cells. After incubation (37 °C, 5% CO_2_, 12 h), the cells were stimulated with LPS and subjected to another incubation step (12 h). Thereafter, levels of IL-1β, IL-6 and TNF-α released from the treated macrophages were detected with an ELISA kit (R&D Systems, Minneapolis, MN, USA). The absorbance was read with an ELISA plate reader (405 nm, with wavelength correction at 650 nm).

### Statistical analysis

The collected data were statistically analyzed by utilizing ANOVA (analysis of variance) with the SPSS software (SPSS/24.0; SPSS Inc., Chicago, IL, USA). The level of significance was shown using the least significant difference (LSD) test. Values are demonstrated as mean ± S.D. of the three independent experiments of each test. “P” values < 0.05 were considered statistically significant.

## Results and discussion

### Chemical synthesis of AuNPs

**Figure 1:**
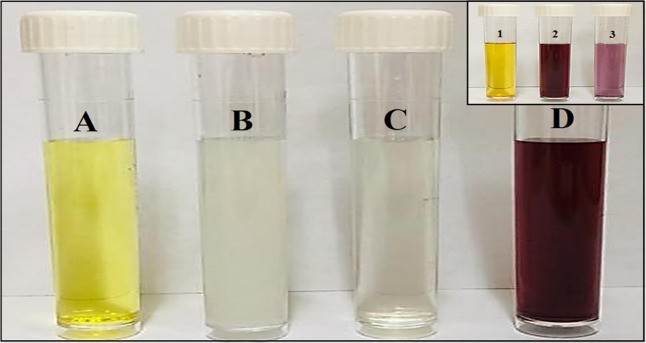
Chemical synthesis steps of gold nanoparticles. (**A**) HAuCl_4_.3H_2_O only (**B**) GSH + HAuCl_4_.3H_2_O (**C**) adjusted pH to 8 (**D**) after added of NaBH_4_.Inset figure shows the chemical reaction of Hsp loaded on AuNPs. The color change of reaction compounds (1) Hsp (2) AuNPs (3) Hsp-AuNPs.

AuNPs were synthesized by three steps. First, the covalent bonding between the cysteine thiolate of GSH with the surface of the gold nanoparticles in the HAuCl_4_.3H_2_O compound. This binding changes the orientation of the terminal polar Glu and Gly residues and exposes them to the anionic carboxylic acids which assist in the solubility and stability of the NPs in water. Cysteine possesses a thiol group (–SH) that is characterized by a high affinity to gold and, consequently, an Au–S covalent bond is formed^27^. In parallel, intermolecular hydrogen bonds are formed among GSH molecules, even during their adsorption to the surfaces of the gold nanoparticles. Hence, these combined molecular mechanisms lead to the aggregation of AuNPs on GSH molecules at certain conditions^27^. The second step includes the adjustment of pH to 8, while the third step includes the formation of AuNPs by the addition of NaBH_4_. The aqueous solution of HAuCl_4_.3H_2_O had a yellow color which was changed into a ruby red color after the addition of NaBH_4_. Figure 1 describes the observation of the reducing agent through the change in color.

### Synthesis of Hsp-AuNPs

GSH is a tripeptide that is naturally composed of thiol, amino and carboxylic groups. The latter group is the reason behind the selection of GSH as a coating agent because it renders AuNPs more stable and water soluble. Furthermore, GSH possesses two free terminal carboxylic acid groups that could be invested to bind to a large spectrum of important molecules in biomedicine. The level of GSH inside the cell is high and, therefore, its modified peptide structure provides good bio-mimicry properties that increase the uptake and bioavailability of nanoparticles^28^. In this study, hesperidin was applied on GSH-coated AuNPs while the pH was maintained at 5, since this acidity level ensures keeping the protonation and the deprotonation properties of the amine and carboxyl groups of Hsp, respectively. Hsp was successfully loaded on AuNPs due to the fact that cationic amine group of Hsp and the anionic carboxyl groups of GSH are electrostatically attracted to each other. The resulting color changes are demonstrated in inset figure at Fig. 1.

### *In vitro* release of Hsp-AuNPs in buffers

**Figure 2:**
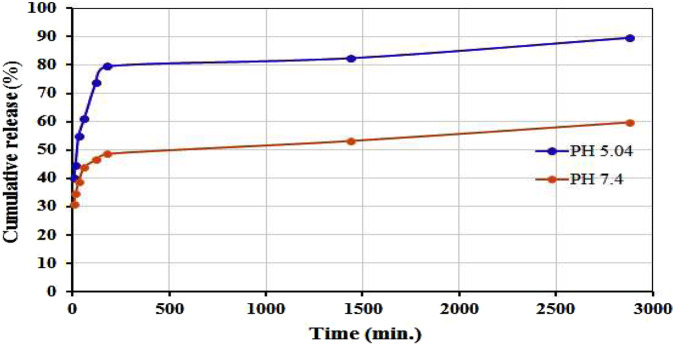
*In vitro* drug release study of Hsp-AuNPs in pH = 7.4 and pH = 5.04.

As shown in Fig. 2, the Hsp release profiles from the Hsp conjugated AuNPs in a releasing medium with pH values of 5.04 and 7.4 was determined using UV-visible spectroscopy assay. After 2880 min, ~89% of the Hsp was released in the pH 5.04 while only ~60% of the Hsp was released in the pH 7.4. However, the release of Hsp from AuNps was faster in acidic pH than in neutral because in an acidic environment the amine group in AuNPs is protonated causing cleavage the bonds between the Hsp and AuNps. Thus, the results show that Hsp-AuNPs were able to particularly stimulate the delivery of hesperidin to cancer cells^29^. Previous studies also reported rapid release of chemotherapeutic drug loaded on AuNPs in acidic conditions when compared to neutral conditions^30,31^.

### UV-VIS spectrum analysis

**Figure 3:**
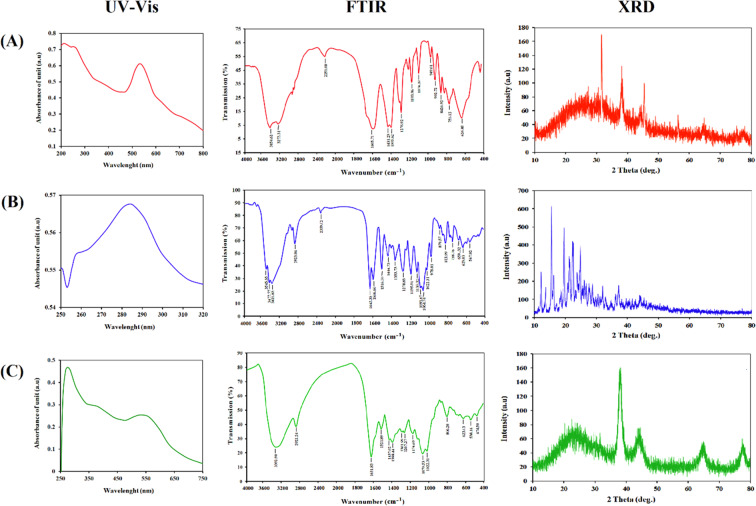
UV-VIS spectroscopy (left panel), Fourier-transform infrared spectroscopy (middle panel) and X-ray diffraction (right panel) analyses for (**A**) AuNPs (**B**) Hsp (**C**) Hsp-AuNPs, respectively.

The current UV-visible spectroscopy results were employed to confirm that the AuNPs were formed and conjugated to Hsp. Figure 3 (left panel) demonstrates the absorption spectra of AuNPs at λmax ~533 nm, caused by the small sized and spherical nanoparticles being excited by the surface Plasmon. While Hsp was a yellow solution that could be seen at 286 nm. The wavelength at maximum absorbance of Hsp-AuNPs demonstrated that they were considerably shifted (λmax ~580 nm) from the unloaded AuNPs condition (λmax ~533 nm), confirming that the drug molecules could be feasibly attached on the surfaces of the AuNPs and that they could alter their photo-physical properties. This attributes to the phenomenon of surface plasmon resonance (SPR) absorption band which is found in noble metals as a result of vibrations that are in tune with the light wave of both electrons in metal NPs^29^. The width of the absorption band and the position of the maximum absorption peak depend on several factors such as the morphology of the particles, aggregation among the particles, dielectric environment and sometimes due to the secondary metabolites responsible for the synthesis^22,32^.

### Fourier transforms infrared spectroscopy analysis (FTIR)

FTIR analysis is essential for the characterization of the functional groups present in the Hsp, AuNPs and Hsp-AuNPs. The FTIR spectra of Hsp, AuNPs and Hsp-AuNPs were recorded in the spectral region 4000–400 cm^−1^ and exhibited in Fig. 3 (middle panel). The FTIR spectra of AuNPs showed the OH symmetric stretching vibration frequency at 3454.62, 3273.31, indicating the presences of alcohol. The presences of the C-H bond was recorded at 2968.55, 2926.11 cm^−1^, whereas the wavenumber 2251.00 cm^−1^ indicated the presence of alkene (C = C) group. The presence of the C = O bond was recorded at 1606.76, 1585.54 cm^−1^. The bands at 1431.23, 1392.65 cm^−1^ belonged to C-C-C stretching, while the wavenumber 1276.92 to 1074.39 cm^−1^ indicated the carboxylic C-O group. These results are compatible with Jiang and his coworkers^33^. The FTIR spectra of Hsp absorption band at 3543.35, 3477.77, 3421.83 cm^−1^ referred to the O-H stretching vibration. The bands at 2928.04, 2359.02 cm^−1^ belonged to the C-H stretch of CH. The bands at 1645.33 cm^−1^ belonged to the C = O stretch, whereas those at 1608.69, 1516.10 cm^−1^ belonged to the C = C stretching of the aromatic group. Absorptions at 1093.67, 1064.74 cm^−1^ referred to the C-O stretch. While, FTIR spectra for Hsp-AuNPs showed many reactions that occurred between groups. The wavenumber 3392.90 cm^−1^ belonged to the hydroxyl group (O-H) stretching vibration, whereas the band at 2922.25 cm^−1^ reflected the alkane (C-H) stretching vibration. The FTIR spectral peak of carbonyl (C = O) stretch was observed at 1631.83 cm^−1^. The bands at 1521.89, 1437.02, 1398.44 cm^−1^ referred to the aromatic (C = C) stretch, whereas the aromatic C-O stretch at was observed at 1301.99, 1267.27, 1174.69 cm^−1^. All the peaks characterizing hesperidin were observed as well in Hsp loaded on AuNPs. These findings are consistent with those of Neha Kiroula *et al*. who demonstrated the possible formation of hydrogen bonds as well as the electrostatic interaction of the protonated amine group with the anionic of AuNPs^34^.

### X-ray diffraction technique (XRD)

The crystal structures of Hsp, AuNPs and Hsp-AuNPs were demonstrated by XRD, as seen in Fig. 3 (right panel). The diffraction peaks of AuNPs are observed at 31.67°, 38.15°, 45.41°, 64.76° and 75.21°, reflecting index values of (111), (200), (220) and (311), respectively, and confirming the polycrystalline face-centered cubic structure. These results are compatible with those of Shamaila *et al*.^29^. The intensity 31.67°, the 2- theta refers to 111 for Glutathione. While, hesperidin displayed the strong characteristic peaks of 2ϴ of 12.22°, 15.59°, 16.27°, 19.62°, 21.31°, 22.57°and 24.81°, indicating its high crystalline structure. When Hsp was loaded on AuNPs, there were peaks observed at 22.46°, 38.12°, 43.99°, 64.58° and 77.42°. According to the data obtained from the XRD analysis, the lack of the effects of the Hsp conjugation reaction on the metallic core size suggests that the observed alterations in the nanoparticles can only be explained by the enlargement of their organic coating^35^.

### Electron microscopy assay

**Figure 4:**
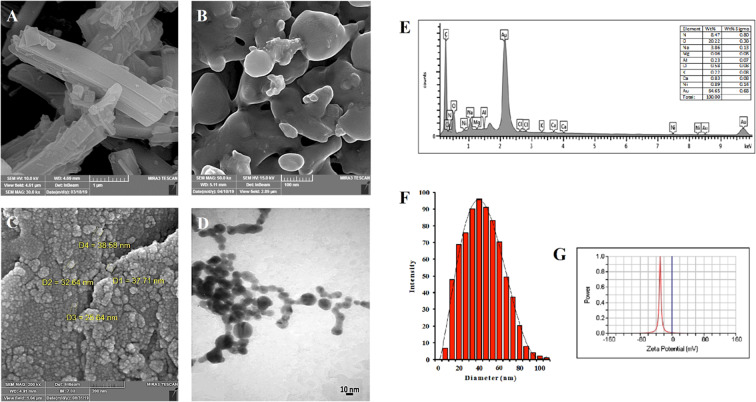
FESEM image analysis of (**A**) Hsp (**B**) AuNPs (**C**) Hsp-AuNPs and (**D**) Transmission electron microscopy (TEM) images of Hsp-AuNPs. (**E**) Elemental analysis by energy dispersive X-ray (EDX) analysis, (**F**) DLS analysis, and (**G**) Zeta potential analysis for prepared Hsp-AuNPs.

For the purpose of studying the particle morphology and imaging of the synthesized Hsp, AuNPs and Hsp-AuNPs, the field emission scanning electron microscopy (FESEM) measurement was performed (Fig. 4A–C). The image of FESEM exhibited relatively a spherical shape of the gold nanoparticle that were formed with a diameter in the range of 15 to 30 nm, while Hsp demonstrated ununiformed shape with aggregation and irregular distribution of the particles in comparison with Hsp-AuNPs that showed a spherical, smooth, and almost homogenous structure. As seen in Fig. 4(D), transmission electron microscopy (TEM) image demonstrates that the Hsp-AuNPs are of a spherical shape, crystalline nature, clearer structural distribution, a smooth surface, and a diameter range of 10 to 50 nm. The results of Hsp clearly reveal that they are homogeneously distributed on the surface of the AuNPs. However, the size of Hsp-AuNPs as acquired by the DLS was slightly larger than that demonstrated by the TEM. This may be attributed to the collective hydrodynamic size of Hsp and AuNPs. Differences in the size of AuNPs measured by TEM versus that measured by DLS suggests coating of AuNPs are coated with certain chemicals that include low- as along with high-molecular weight flavonoids^36,37^.

### Energy dispersive X-ray spectroscopy (EDAX)

EDAX is a method that is applied to analyze the elements of samples and to characterize them chemically. The main principle is based on the sample being excited by a source of X-ray. Its characterization ability is mainly attributed to the fact that each element possesses a specific atomic structure that allows a specific group of peaks on its spectra of electromagnetic emission^38^. EDAX analysis for Hsp-AuNPs shows the presence of Au (64.65%) as the major element in the sample, in addition to carbon, oxygen and other elements. Further, the percentage composition of carbon and oxygen demonstrated an elevation in the drug-loaded AuNPs (Fig. 4E), confirming the loading of hesperidin loaded on the AuNPs. The high content of carbon, along with the low oxygen content, within the sample demonstrated the diverse physiochemical characteristics of AuNPs as a biocapping agent.

### Zeta potential analysis and particle size analysis

To further characterize the Hsp-AuNPs, zeta potential and particle size were measured using the zetasizer machine. The size of Hsp-AuNPs averaged about 40 nm (Fig. 4F), while the zeta potential reading was −29.40 mV (Fig. 4G), indicating that the loaded nanoparticles were stable in solution, as described by the guideline^39^. Zeta potential is an essential physicochemical indicator of the stability of nanosuspensions. Values of extreme positivity or negativity result in stronger repulsive forces, while repulsive forces among particles that have similar charges lead to the prevention of particles aggregation, thereby ensuring easy re-dispersion^39^. In general, potential values exceeding 30 mV confer good stability, while those exceeding 60 mV confer excellent stability^40^. A value of 20 mV confers short-term stability, whereas those ranging between −5 mV and 5 mV demonstrate fast aggregation. Particle’s surface charge and the nature of the binding of the drug with the NPs are the essential factors determining the rate at which the drug desorbs in the NP as well as the efficiency of loading. ZP value can also be employed to indicate if the encapsulation of a charged active material occurs within the center of the NP or on its surface^41^. DLS analysis is applied for the measurement of how thick is the shell of a capping or stabilizing agent that envelops the metallic NPs as well as the actual size of the metallic core. The comparatively large size of Hsp-AuNPs that was observed using DLS indicated the attachment of the drug on the surface of gold nanoparticles. *In vivo* bio-distribution proposed that the slightly negative and 150 nm-sized nanoparticles had the tendency of more efficient accumulation in tumor cells. These findings can be employed as a guideline for the rational designing of drug nanocarriers that have maximum therapeutic efficiency and *in vivo* predictable characteristics, where controlling the particle size and surface charge is a significant matter. Cationic NPs have the parallel benefits of being efficient carriers for drugs as well as the capability of making complexes with the nucleic acids and functioning as gene carriers. An increased amount of researches have been currently employing the cationic NPs due to their safety and efficiency in carrying therapeutic materials^42^.

### *In vitro* hemolysis assay

**Figure 5:**
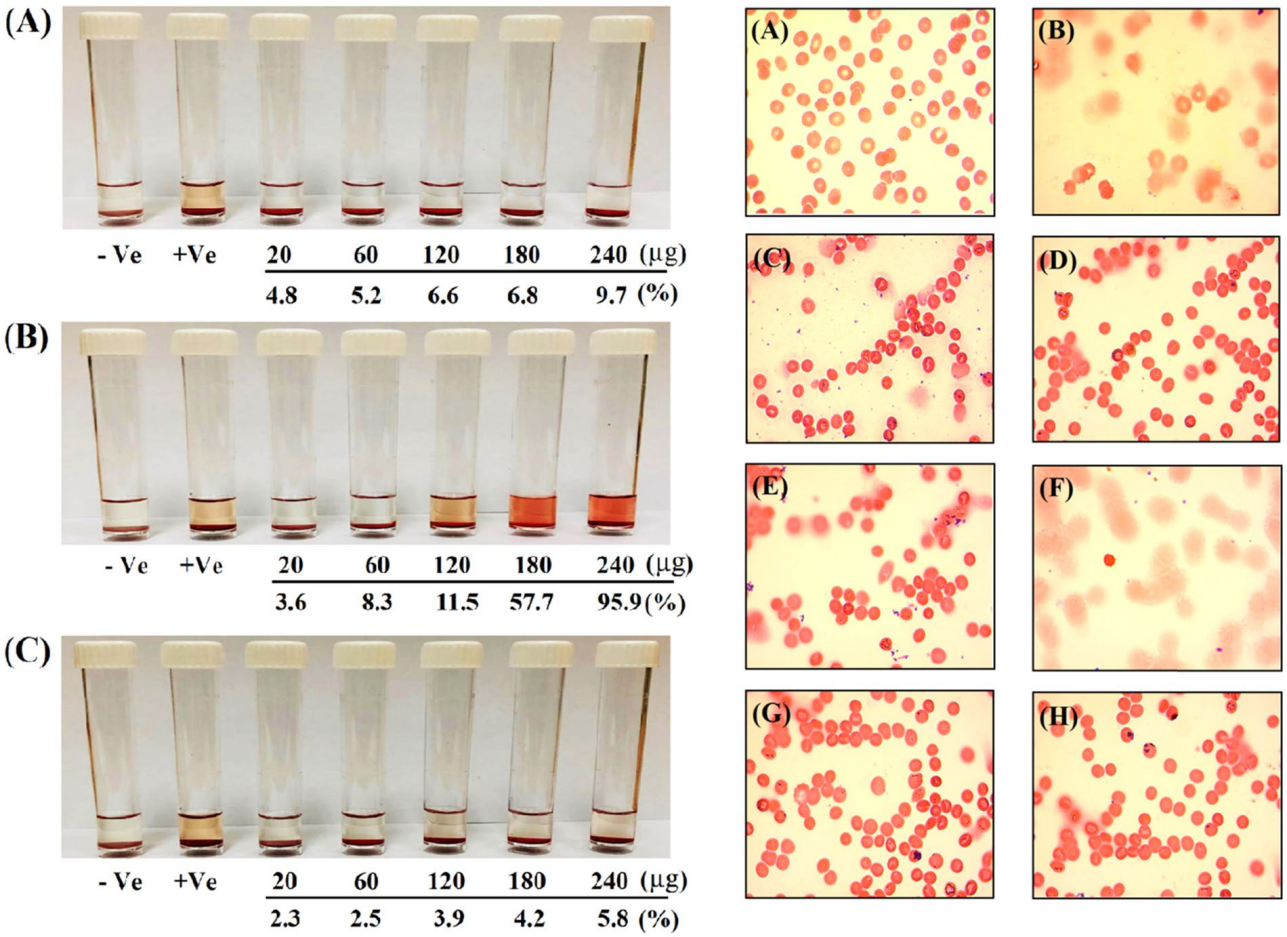
Photograph of human RBCs (Left panel) showing percentage of blood hemolysis on incubation with (**A**) AuNPs (**B**) Hsp (**C**) Hsp-AuNPs at 20, 60, 120, 180 and 240 µg mL^−1^. Light microscopic images (Right panel) show the hemolytic toxicity of AuNPs (**C** and **D**), hesperidin (**E** and **F**) and Hsp-AuNPs (**G** and **H**) at 20 and 240 µg mL^−1^ along with the negative (**A**) and positive (**B**) controls.

The images of RBCs following exposure to Hsp, AuNPs, Hsp-AuNPs are shown in Fig. 5 (left panel). It was apparent that the percentage of hemolysis with hesperidin was higher than that observed with AuNPs and Hsp-AuNPs. The haemolysis effects of Hsp-AuNPs at the concentrations of 20, 60 and 120 µg mL^−1^ were consistent with the acceptable limits of less than 3.9%. While, the concentrations of 180 and 240 µg mL^−1^ resulted in a hemolysis percentage of lower than 6% in comparison to the similar concentrations of Hsp or AuNPs. Based on the rules of the ASTM E2524-08 standard, a haemolysis value that is higher than 5% reveals that the tested nanoparticles caused damage to RBCs^43^. The same standard states that AuNPs and Hsp-AuNPs are haemocompatible with the possibility of their usage within the body, but in low concentrations only. Figure 5 (right panel) shows the results of the light microscope examination of RBCs with the influences of low and high concentrations of Hesperidin, AuNPs, Hsp-AuNPs. The haemolysis test revealed that the morphology of the RBCs was not damaged by the lower concentration range of AuNPs or Hsp-AuNPs, However, the Hsp-AuNPs had a lower hemolytic activity in this regard, while the pure Hsp demonstrated stronger damaging effects to these cells, represented by destroying their cell membrane. According to study performed on hyaluronic acid coated poly (butyl cyanoacrylate) nanoparticles, the interplay of the RBC with the synthetic polymer was predominantly controlled by the softness of the NP surface. It is considered that if the nanoparticles cover with synthetic polymers, they are not recognized as foreign materials by biological cells, if the polymer surfaces are soft and hydrophilic and in this way one can decrease the hemolytic potential^44^. Non-specificity was demonstrated to be associated with the mechanisms of direct hymolysis actions of various toxic compounds. In particular, xenobiotic plant derivatives like phenols exert the capability of inducing haemolysis by oxidizing haemoglobin, leading to the formation of metahemoglobin^45^. Our results are highly consistent with those of Sathishkumar *et al*. who demonstrated that chrysin AuNPs are well compatible with human RBCs^46^. Another study revealed that AuNPs synthesized by *Z. officinale* extract are highly compatible blood cells without initiation of cell aggregation or activation of platelets^47^. The above studies on RBCs suggest that the synthesized Hsp-AuNPs in the present study is potentially biocompatible and can be safely used inside the body.

### *In vivo* toxicity assays

**Figure 6:**
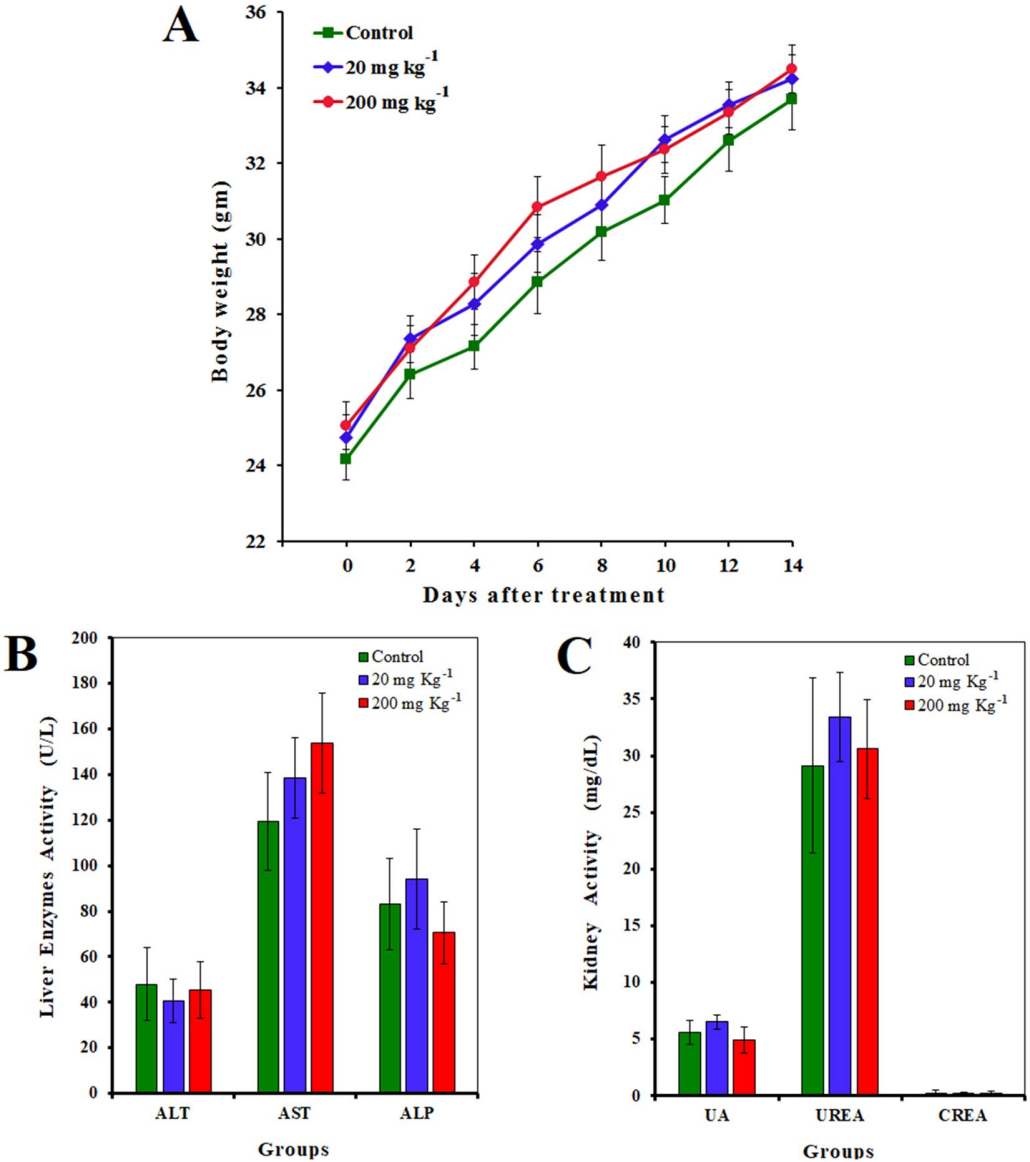
(**A**) Body weight changes for mice treated with Hsp-AuNps at doses of 20 mg kg^−1^ and 200 mg kg^−1^ by using intraperitoneal injection. Body weight was measured every two days. Each point represents the mean ± S.D. of 3 mice. (**B,C**) Biochemical results (urea, creatinine, uric acid, ALT, AST and ALP) of mice treated with Hsp-AuNps. Data were analyzed by the differences between the doses and control group.

Figure 6; A demonstrates differences in the body weight of mice following the treatment with two concentrations of Hsp-AuNPs. It can be observed that treatment with Hsp-AuNPs in the dose range of 20–200 µg kg^−1^ for 14 days did not cause mortality. In addition, differences (P ≤ 0.05) in body weight were insignificant between the Hsp-AuNPs-treated mice and control mice. Moreover, no abnormal clinical signs or behavior were detected between the treated and the untreated mice. Taken together, Hsp-AuNPs treatment apparently did not cause any toxicity in the animals, while adverse behavioral reactions were not observed too. No alterations in the macroscopic organs were observed in the treated mice when the necropsy examination was performed at the end of the experiments. A previous study showed that intraperitoneal injection of AuNPs at low concentrations has no remarkable toxic effects even following their gradual breakdown *in vivo*. Also, higher doses of AuNPs induced insignificant reductions in the weight of animals^48^. Nevertheless, it is still necessary to investigate the toxic effects, including those on body weight, of various administration routes and particle sizes.

**Figure 7:**
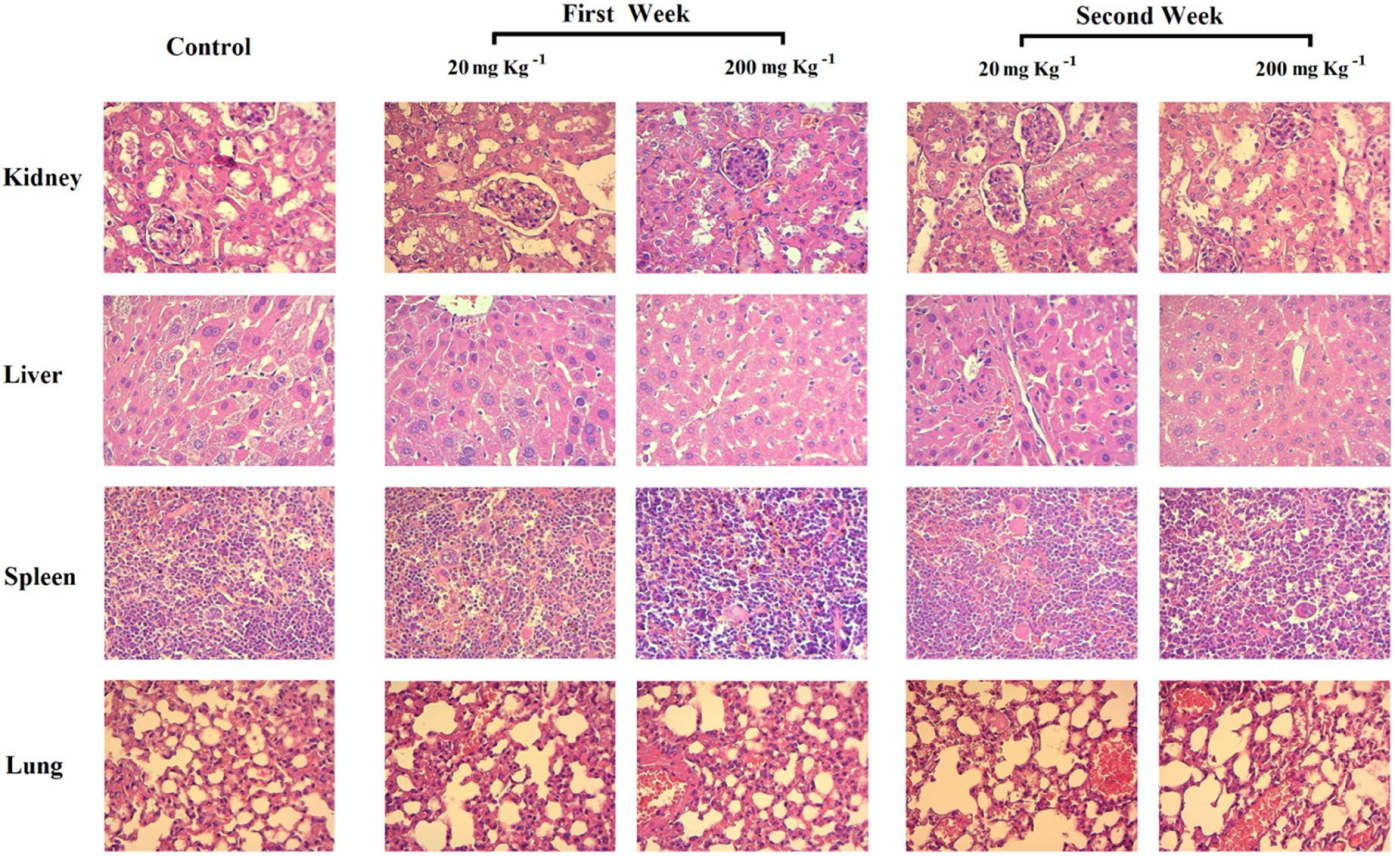
Histological images for kidney, liver, spleen and lung of mice treated with Hsp-AuNps by using administration intraperitoneal injection by different doses (20 mg kg^−1^, 200 mg kg^−1^).

It is essential to study if Hsp-AuNPs result in toxic effects in organs such as the liver and kidney. Therefore, we presented the biochemical data from the serum of the stdies mice, such as,alanine transaminase (ALT), aspartate transaminase (AST), blood urea nitrogen and creatinine in Fig. 6B. Urea and creatinine are metabolic products correlated with the functioning of the kidney (Fig. 6C). The serum concentrations of ALT, AST and ALP were measured to indicate the effects on liver function. For these parameters, the differences caused by the treatment were insignificant (P ≤ 0.05). Moreover, we measured uric acid concentrations and showed a non-significant reduction (P ≤ 0.05). However, of the reduced serum level of uric acid (hypouricemia) is a known indicator of drug toxicity^47^. Also, levels of total bilirubin, total protein, and albumin, which are also markers of the liver function, were not different to the control group (data not shown). Histological examinations were carried out to observe if the two doses of Hsp-AuNPs could lead to toxic effects such as tissue damage, inflammation or lesions. Histological imaging of the liver, spleen, lung and kidney, was conducted to investigate such potential toxicity. Figure 7 reveals no apparent damaging effects and histopathological abnormalities in mice treated with Hsp-AuNPs.

### MTT assay

**Figure 8:**
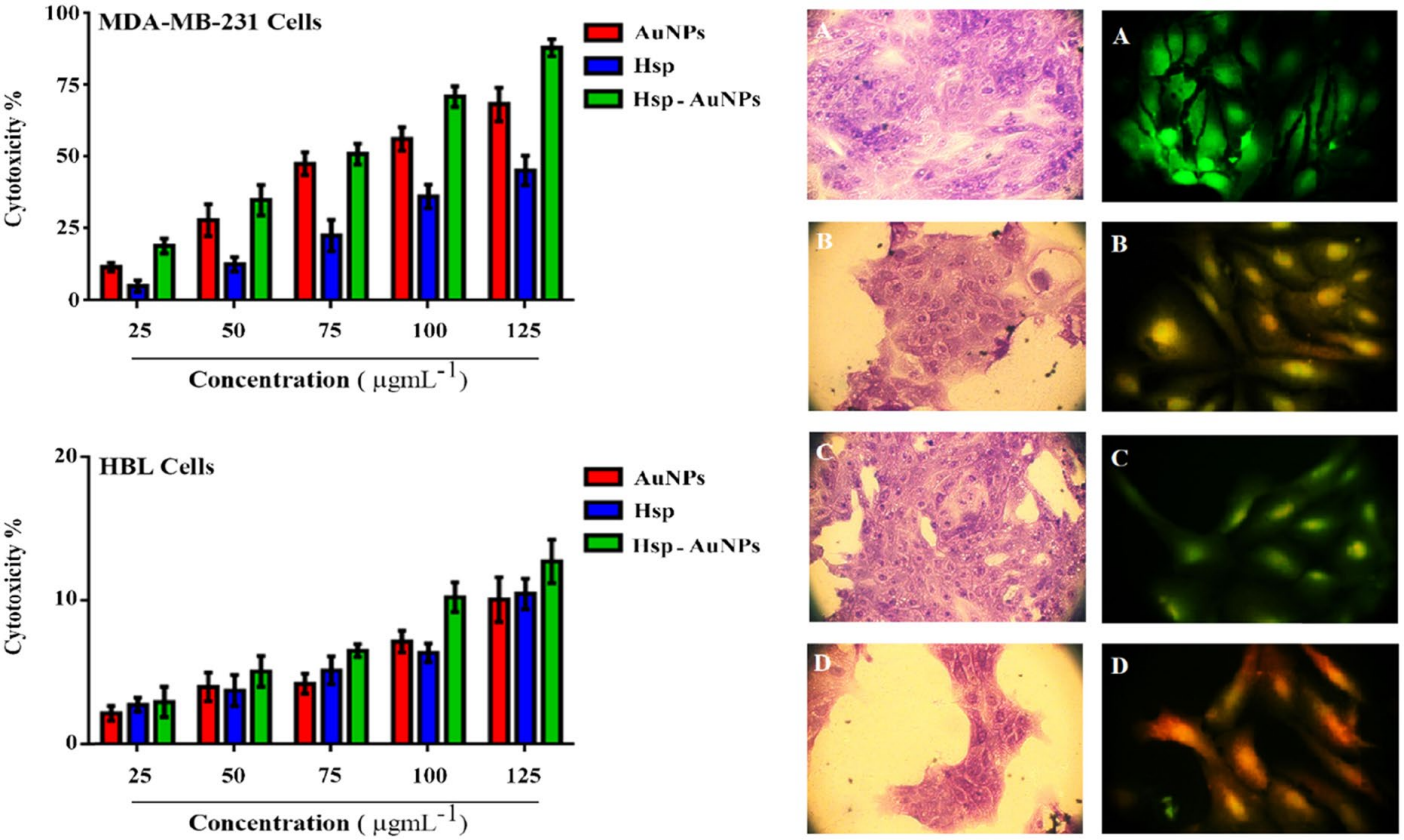
Histogram for growth inhibition of breast cancer MDA-MB-231 and normal human HBL cell lines treated with different concentrations (25, 50, 75, 100 and 125 μg mL^−1^) of Hsp, AuNPs and Hsp-AuNPs (left panel). While, right panel represents a microscopic images of MDAMB‐231 cell line after treatment with IC_50_ of Hsp, AuNPs and Hsp-AuNPs and stained with crystal violet dye and AO/EtBr, respectively. (**A**) non-treated cells (**B**) cells treated with Hsp (**C**) cells treated with AuNPs (**D**) cells treated with Hsp-AuNPs.

Cell viability results using MTT test provide clear explanations for the mechanisms taken by the cells to respond to a toxic agent. In the present study, the synthesized Hsp-AuNPs exhibited a higher anticancer activity to MDA-MB-231 breast cancer cells as compared to hesperidin or AuNPs, with a dose-dependent cellular toxicity. Hsp-AuNPs exhibited higher cytotoxic effects, followed by AuNPs and Hsp. The highest cytotoxic effect (88%) was observed at a dose of 125 µg mL^−1^ of Hsp-AuNPs, while at 25 µg mL^−1^ 19% cells were affected, as compared to those of the control. Treatment with AuNPs caused 68.3% and 11.6% toxicity, whereas that of hesperidin caused 45.3% and 5% toxicity, respectively. While, no such activity was seen in human normal breast epithelial cell line (HBL-100). The proliferation rate of normal cell demonstrated less cytotoxic effect when compared with those observed in cancer cells (Fig. 8). The cytotoxic effects of the formulated Hsp-AuNPs were much stronger than those of free hesperidin, indicating the improvement in the anticancer efficiency of hesperidin after being functionalized with AuNPs. It is necessary to note that the concentration of the synthesized Hsp-AuNPs employed in this case was very lower than that of hesperidin. This reduction in cell viability with raising NPs concentration indicates a higher number of NPs that were able to accumulate within the cells, causing them stress and eventually death. Factors as size, morphology, surface area and surface functionalization are the main players affecting bio-kinetics and toxicity^49^. It was demonstrated in a recent study that treatment with hesperidin nanoparticles resulted in a decrease in cell proliferation and a significant inhibition of growth of human breast carcinoma cells (MCF-7) (p ≤ 0.05) in a concentration-dependent manner^21^. Another study demonstrated the toxic effects of LIN-AuNPs against human breast cancer MCF-7 cells, as indicated by inducing apoptotic cell death in a dose depended manner^50^. The present results suggest that the Hsp-AuNPs-associated cell death might possibly occurred due to apoptosis or necrosis. There is a need to investigate the mechanisms followed by the Hsp-AuNPs to induce cell death.

### Crystal violet assay against MDA-MB-231

The cytotoxicity impact of Hsp-AuNPs (IC_50_ concentration) on MDA-MB-231 cells was additionally validated by employing a crystal violet test, as illustrated in Fig. 8 (left panel). The results revealed that hesperidin alone was lesscytotoxic to MDA-MB-231 cells in the tested concentration, and was less effective as compared with AuNPs. Conversely, Hsp-AuNPs proved to have significantly higher toxicity to MDA-MB-231 cells. The Hsp-AuNPs stimulated various morphological changes in the cells that also formed clusters with lower numbers of extensions and impaired cell-cell communication, while control cells lacked these kinds of alterations. The small size of NPs enables them to show more efficient penetration into the cells compared to micro-particles, making it easier to deliver higher amounts of the drug and to achieve better results. Specifically, AuNPs have biological functions against various damaging reactions such as oxidation, inflammation, angiogenesis, and cancer^51,52^. Furthermore, AuNPs have the properties of being bio-compatible and non-toxic, making them preferable drug carriers^53^. Previous investigations revealed that Hsp exerts remarkable cytotoxicity towards selected human cancer cell lines such as those of the cervix, liver, larynx, and breast^23,54^. Moreover, it was demonstrated that Hsp exerts chemo-preventive activities along with apoptotic impacts that lead to the inhibition of colon carcinogenesis in mice^55^. In line with this, our findings suggest that hesperidin can be employed in the treatment of cancer, since it showed higher cytotoxic effects to tumor cells when it was conjugated to AuNPs.

### Double staining with acridine orange –ethidium bromide (AO/EtBr)

Apoptosis is a special and essential form of programmed cell death involving the removal of abnormal cells during the development and homeostasis of mammalian tissues. Induction of apoptosis is regarded as a standard and preferable approach in cancer treatment. Apoptosis has a variety of characteristic hallmarks as those observed when the chromatin is condensed, the nucleus is fragmented, the cells shrink, and the membranes bleb^56^. The present study employed acridine orange/ethidium bromide (AO/EtBr) dual staining along with fluorescence microscopy to validate the observed alterations in the shapes of the nuclei of the treated MDA-MB‐231 cells (Fig. 8; left panel). Cells treated with Hsp-AuNPs exerted extensively higher damages to membrane integrity in comparison to untreated cells. Fewer effects were observed in cells treated with hesperidin or AuNPs (Fig. 8; left panel). Nuclei of the untreated cells had a normal structure the formed bright green color showed stability, whereas stimulated alterations, including chromatin condensation, were clearly observed in the treated cells. Apoptotic cells have red to orange nuclei in addition to various degrees of condensation or fragmentation. However, early apoptotic cells exerted a yellow color while the late ones had nuclei that exerted condensation and a red color. The changes in the morphology of cells treated with Hsp-AuNPs reveals the induction of cell death by apoptotic but not necrotic mechanisms. These results confirmed the significant induction of apoptotic mechanisms in breast cancer cells by Hsp-AuNPs rather than hesperidin or AuNPs alone. Consistent with the present findings, Bhat *et al*. demonstrated that hesperidin was able to induce apoptosis in prostate cancer cells^57^. One previous investigation suggested that increasing intracellular ROS production can be, at least partially, responsible for the killing activity exerted by the anticancer drugs^58^. Incubation of cancer cells with nanoparticles was associated with altered cell proliferation and enhanced apoptosis under potent influences from alterations in levels of ROS production. Nevertheless, events such as enhanced apoptotic morphological alterations and DNA damage could be possibly caused by elevated ROS concentrations and the associated altered mitochondrial membrane^59^.

### Tumoricidal activity of AuNPs-Hsp

**Figure 9:**
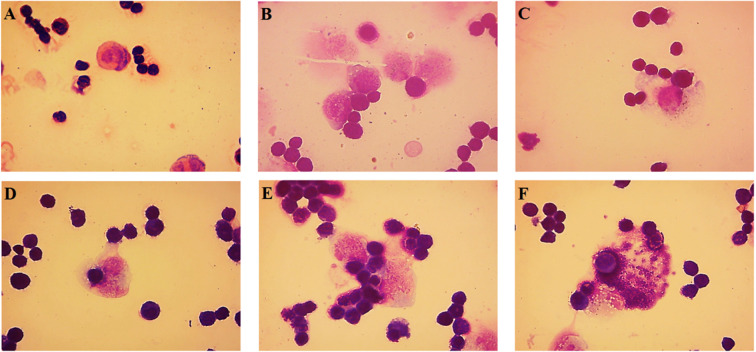
Effect of Hsp-AuNPs on the functional activity of macrophages to tumor cells and apoptotic process. Cells obtained from ascites fluid on the 14th day of tumor growth after treatment with Hsp-AuNPs. Stages of phagocytosis are represented.

Figure 9 demonstrates the distribution of macrophages which had increased sizes and larger contents of cytoplasmic vacuoles. The treatment with Hsp-AuNPs induced higher functional activities of macrophages in Ehrlich ascites tumor cells -bearing mice compared with control group. The observed higher activities against tumor, likely due to the Hsp-AuNPs, are possibly related to an elevation in the activities rather than the numbers of these cells in the peritoneum, leading to slowing the growth of tumor. It is widely accepted that MN cells, prominently macrophages, are the major contributors in tumor rejection. The various patterns of macrophage spreading demonstrated the effects of Hsp-AuNPs on the functional states of these cells. These results show that the induction of macrophages by Hsp-AuNPs can be the most powerful effector anticancer mechanism of the tested compounds *in vivo*. These data propose that Hsp-AuNPs possibly has a direct interference with the growth of Ehrlich ascites tumor cells upon the early stage of treatment, resulting in a remarkable elimination of these cells. Thus, Hsp-AuNPs might exert direct and/or indirect activities on cancer cells through the induction of host cells, mainly macrophages, as well as the stimulation of the secretion of various cytokines, including IL-1, IL-6, IL-8, TNF-α and NO. The capability of macrophages to show responses to NPs also varies based on their activation state. Nanomaterials are able to stimulate the release of certain toxic mediators, including reactive oxygen species (ROS), as these materials have the potential of direct entry to the mitochondria, leading to their activation of macrophages and induction of inflammatory pathways. Morishige *et al*. and Yazdi *et al*. revealed the responsibility of ROS, stimulated by Hsp-AuNPs, for the induction of inflammasomes, resulting in the maturation of IL-1β maturation in macrophages^60,61^. A number of such e cytokines can confer direct cytotoxicity against cancer cells, whereas others exert actions against other types of cells such as natural killer cells and cytotoxic T lymphocytes. Furthermore, these cytokines can possibly enhance the synthesis of C-reactive protein and complement factor C3 could function as opsonins on tumor cells^62^. A mixture of such activities could lead to the inhibition and removal of tumor cells. However, the mechanism of tumor cell death that is associated with cytokines production is not clearly understood. Thus, in order to provide better understanding for anti-tumor activities, an extended array of experiments were performed in this study.

### Pro-inflammatory cytokines

**Figure 10:**
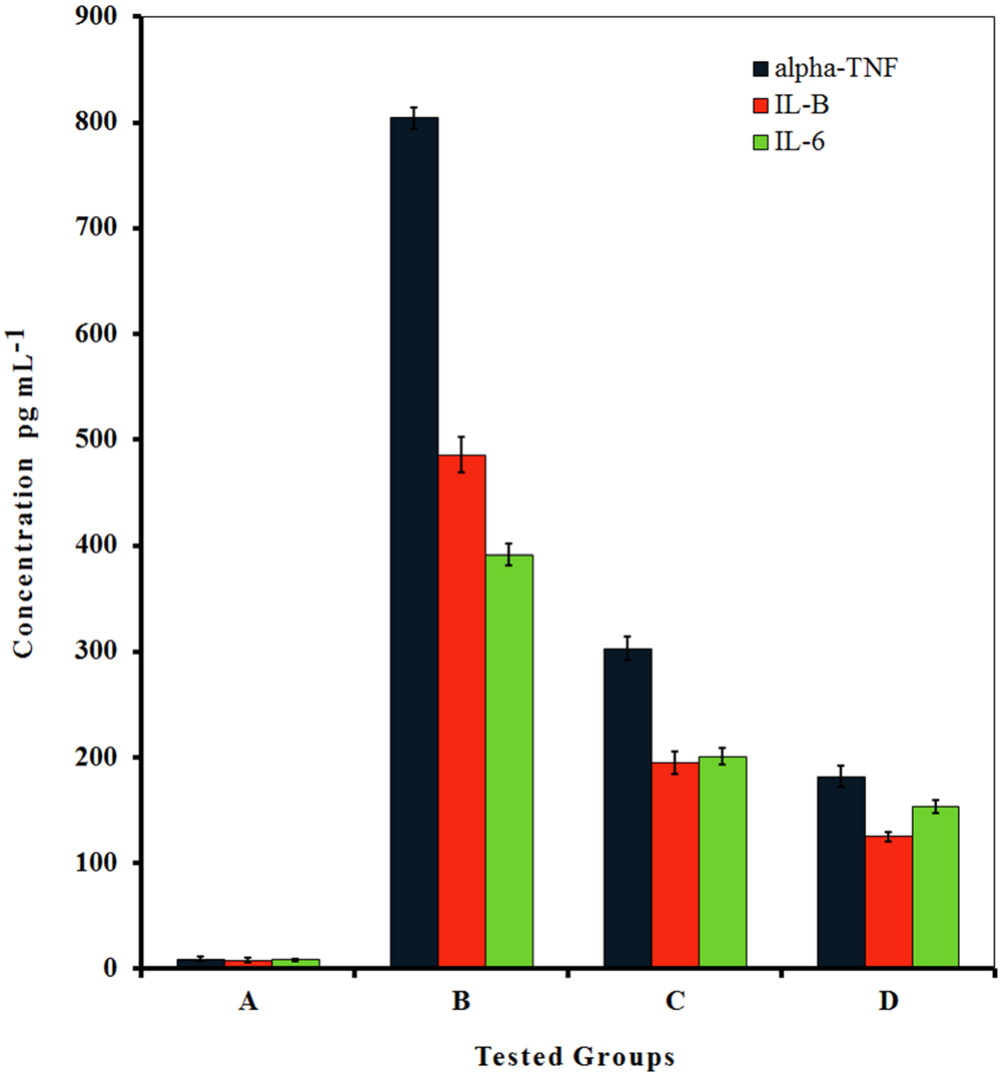
Effect of Hsp-AuNPs on IL-1β, IL-6 and TNF-α production of bone marrow derivative macrophage cells. The cells were treated with two concentrations of Hsp-AuNPs (50, 250 µg mL^−1^) for 12 h and then activated with LPS and ATP for further 12 h. (**A**) Non treated macrophage cells, (**B**) macrophage cells treated with LPS, (**C,D**) macrophage cells treated with Hsp-AuNPs at 50, 250 µg mL^−1^ respectively. Data are reported as means ± S. D. from three independent experiments.

As shown in Fig. 10, the production of IL-1β, IL-6 and TNF-α was assessed in bone marrow derivative macrophage cells exposed to 50 or 250 µg mL^−1^ of Hsp-AuNPs. The results provide a clear indication that Hsp-AuNPs significantly attenuated the production of all of the aforementioned cytokines, as compared to that induced by LPS only, in a concentration-dependent manner. The current findings provide additional evidence that supports the anti-inflammatory properties of Hsp-AuNPs in different disease models. Previous investigations demonstrated that AuNPs up-regulated cytokine expression in other cell types^63^. While, other findings showed that hesperidin inhibited the production of pro-inflammatory cytokines as TNF-*α* and IL-1*β* in cerebral ischemia in addition to IL-8, IL-6, IL-12 and vascular cell adhesion molecule 1 (VCAM-1) in acute lung inflammation induced by LPS *in vivo*^64^. Barbarisi *et al*. found that boswellic acid, a natural product, significantly increased the anticancer potentials of antitumor drugs (TMZ and Afatinilib) in human glioblastoma cells. These potentials were related to it’s anti-inflammatory, and antioxidant activities, based on inhibition of growth factors and pro-inflammatory interleukins^65^.

Previous studies performed on nanocarrier of quercetin combined with anticancer drugs such as temozolomide and macrolide showed specificity and efficacy of these drugs in human glioblastoma and breast cancer treatment, respectively. The combination with anticancer drugs provoked cell cytotoxicity, antioxidants and anti-inflammatory potentials as compared to unformulated quercetin. Nanohydrogel of quercetin was able to reduce significantly IL-8, IL-6 and VEGF production in pro-inflammatory conditions^66,67^.

Dissection of the mechanism(s) involved in the pro-inflammatory response is commonly challenged by the presence of other materials in the nanoparticle structure. For instance, fibrous TiO_2_ nanoparticles could not cause cytokine induction until their combination low, unreactive amounts of endotoxin, where they strongly induced cytokines of the IL-1 family (IL-1β, IL18, IL33) via a cathepsin B-mediated pathway^68^. Another often observed problem is the production of unwanted cytokines due to the chemical impurities present in engineered NPs. For instance, early investigations dealing with carbon nanotubes revealed their ability of the induction of pro-inflammatory cytokines, an impact that could be reversed by the purification of these NPs from iron impurities^69^. Such complications in investigating the pathways responsible for the inhibition of cytokines production upon treatment of cells or animals with nanomaterials warrant further research.

## Conclusions

In the present study, the Hsp-AuNPs were produced by employing a simple and efficient approach that included several physico‐chemical methods. These NPs demonstrated anti-oxidant activities as well as protective effects against DNA damage resulting from H_2_O_2_. The findings revealed that Hsp-AuNPs was cytocompatible and do not cause appreciable toxicity or adverse behavioral reactions in mice, implying its safety in biomedical applications. Furthermore, this study emphasized that the Hsp-AuNPs possess strong cytotoxicity and enhancement of apoptosis in human breast cancer cells. The tumor growth in EAT-bearing mice was effectively inhibited by the tumoricidal activity of Hsp-AuNPs through inducing the functional activity of macrophages. The effect of Hsp-AuNPs may be well utilized as an efficient strategy in clinical cancer therapies and can be explored to a greater extent for drug delivery applications. Currently available therapies are inadequate and needs to be supplemented with novel targeted therapies to tackle the tenacious tumor. Thus, this investigation reveals that the prepared particles may be applicable to the multidrug nanoformulations with anticancer drugs for breast cancer therapy. Designing nanoformulations that can adequately penetrate all of these sites without causing excessive adverse effects is of critical importance.
